# Distinct Molecular Mechanisms Characterizing Pathogenesis of SARS-CoV-2

**DOI:** 10.4014/jmb.2206.06064

**Published:** 2022-08-23

**Authors:** Su Jin Lee, Yu-Jin Kim, Dae-Gyun Ahn

**Affiliations:** Department of Convergent Research of Emerging Virus Infection, Therapeutics and Biotechnology Division, Korea Research Institute of Chemical Technology, Daejeon 34114, Republic of Korea

**Keywords:** COVID-19, coronavirus, SARS-CoV-2, mechanism, pathogenesis

## Abstract

The severe acute respiratory syndrome coronavirus 2 (SARS-CoV-2) pandemic has continued for over 2 years, following the outbreak of coronavirus-19 (COVID-19) in 2019. It has resulted in enormous casualties and severe economic crises. The rapid development of vaccines and therapeutics against SARS-CoV-2 has helped slow the spread. In the meantime, various mutations in the SARS-CoV-2 have emerged to evade current vaccines and therapeutics. A better understanding of SARS-CoV-2 pathogenesis is a prerequisite for developing efficient, advanced vaccines and therapeutics. Since the outbreak of COVID-19, a tremendous amount of research has been conducted to unveil SARSCoV-2 pathogenesis, from clinical observations to biochemical analysis at the molecular level upon viral infection. In this review, we discuss the molecular mechanisms of SARS-CoV-2 propagation and pathogenesis, with an update on recent advances.

## Introduction

Since the first coronavirus-19 (COVID-19) case was reported in Wuhan, China in 2019, the pandemic has accounted for more than 530 million infections and nearly 6.3 million deaths (as of June 24, 2022) [1]. Owing to tremendous efforts to overcome the COVID-19 pandemic, several effective vaccines and antiviral drugs were developed and are currently being used. However, even two years after the outbreak, severe acute respiratory syndrome coronavirus 2 (SARS-CoV-2), the causative agent of COVID-19, poses a threat to global health as its variants have rapidly emerged with genetic mutations enhancing viral infectivity, disease severity, and ability to evade host immunity [2, 3].

Coronavirus infection begins with specific binding of the coronavirus spike (S) protein to cellular entry receptors identified for several coronaviruses, including aminopeptidase N (APN) for human coronavirus 229E (HCoV-229E); angiotensin-converting enzyme 2 (ACE2) for HCoV-NL63, SARS-CoV, and SARS-CoV-2; dipeptidyl peptidase 4 (DPP4) for Middle East respiratory syndrome coronavirus (MERS-CoV); and 9-O-acetylated sialic acid (9-O-Ac-Sia) for HCoV-OC43 and HCoV-HKU1 [4-6]. Interactions between viral S proteins and their specific cellular receptors are important determinants of the host range of infection, tissue tropism, and viral pathogenesis [7]. During the viral life cycle, coronaviruses express viral proteins and generate new genomic RNAs, producing infectious progeny viruses harboring a remarkably large RNA genome, reaching up to ~30 kilobases [8]. Its RNA genome contains untranslated regions (UTRs) at the 5' and 3' ends, where cis-acting secondary RNA structures are located, which are essential for RNA synthesis [9]. Two-thirds of the genome consists of two large open reading frames (ORF1a and ORF1b) at the 5' end, which translate the polyproteins pp1a and pp1ab. These polyproteins are cleaved by viral proteases, papain-like protease (PL^pro^) and 3C-like protease (3CL^pro^, also known as the main protease), generating 15–16 nonstructural proteins (nsps). These viral proteins are necessary for viral RNA synthesis and modification, and form a replication and transcription complex (RTC)[10]. RTC synthesizes new viral RNA copies and generates an extensive set of 3'-co-terminal subgenomic RNAs (sgRNAs), which mainly express viral structural proteins necessary for virion assembly: nucleocapsid (N), membrane (M), spike (S), and envelope (E) proteins. In many cases, these viral proteins and other accessory proteins play additional roles in evading host immune responses and act as virulence factors involved in viral pathogenesis upon viral infection [11, 12].

Although coronaviruses share similar viral genome structures and replication mechanisms, the viral proteins of each coronavirus feature enormous genetic diversity, depending on the virus lineages and their hosts. Coronaviruses have evolved continuously over the past 1,000 years and were first identified in animals [13]. The first identified coronaviruses were infectious bronchitis virus (IBV) in chickens in 1947 [14] and mouse hepatitis virus (MHV) in mice in 1949 [15]. Since the discovery of the first human coronaviruses (HCoV-229E and HCoV-OC43) in the 1960s [16, 17], significant research has been conducted to understand the basic principles of the coronavirus life cycle and pathogenesis. Subsequent coronavirus studies have accelerated since the emergence of SARS-CoV in 2002 [18] and MERS-CoV in 2012 [19], broadening the view of coronavirus as a zoonotic pathogen causing serious diseases in humans. Bats are believed to be reservoirs of various CoVs including SARS-CoV-2 [13, 20], and MERS-CoV-infected dromedaries can transmit to humans. These zoonotic diversities have endowed coronaviruses with distinct molecular features, including various receptors.

The COVID-19 pandemic has encouraged research on the unknown and unique features of SARS-CoV-2, which has helped to build up extensive knowledge about this emerging virus. However, the specific molecular functions of the viral proteins and precise mechanisms of viral pathogenesis are not yet fully understood. In this review, we discuss the molecular mechanisms of SARS-CoV-2 propagation, pathogenesis, and describe how virus–host interactions differ depending on the type of virus. In particular, we explored recent clinical and experimental advances to better understand SARS-CoV-2 pathogenesis.

### Receptors and Tissue Tropism of SARS-CoV-2

The entry of coronaviruses is mediated by the S protein, which binds to host receptors, thus attaching to the cell membrane and causing subsequent membrane fusion. Generally, receptor recognition differs depending on virus subtype. In case of SARS-CoV-2, ACE2 is a key receptor that interacts with the receptor-binding domains (RBDs) of viral S protein [21, 22]. SARS-CoV and HCoV-NL63 also utilize ACE2 as their main host cell receptor [23, 24]. APN is another receptor for several types of coronaviruses such as HCoV-229E, transmissible gastroenteritis coronavirus (TGEV), and porcine respiratory coronavirus (PRCV) [25-27]. In addition, MERS-CoV uses dipeptidyl peptidase-4 (DPP4) as its main receptor [28, 29].

**Fig. 1:**
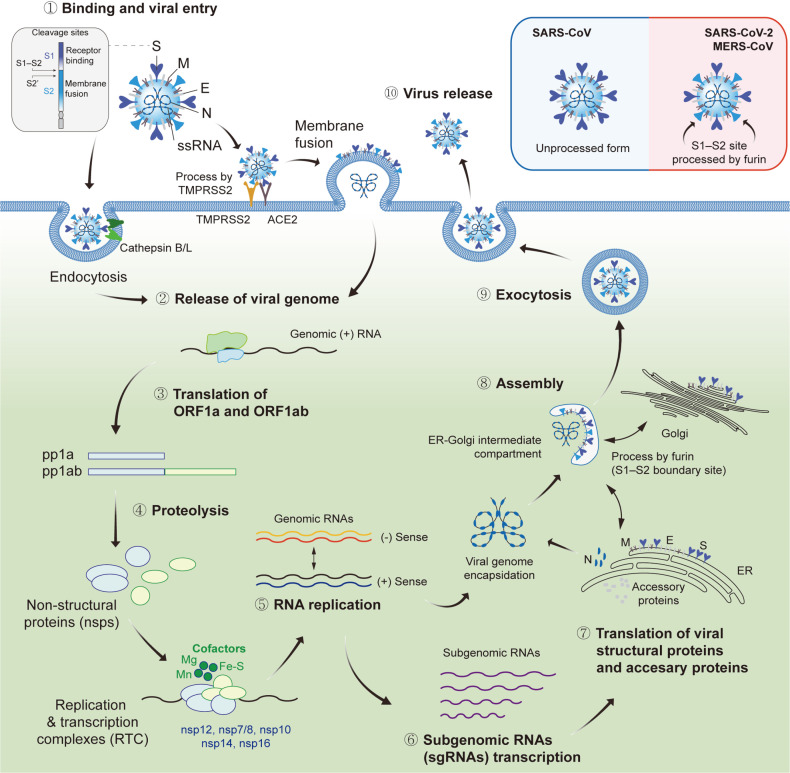
Replication of coronaviruses. The coronavirus is an enveloped virus with a positive sense, single-stranded RNA genome, which is encapsidated by nucleocapsid (N) protein. Its virion consists of spike (S), envelope (E), membrane (M), and N proteins. The entry of coronaviruses is mediated either by clathrin-mediated endocytosis or by membrane fusion facilitated by their receptors: ACE2, TMPRSS2, and cathepsin B/L (①). The released viral genome (②) is translated generating viral polyprotein pp1a and pp1ab from two open reading frames (③); ORF1a and ORF1b, which encode non-structural protein (nsp) complexes 1-11 and nsp12-16, respectively. The pp1a and pp1ab are then processed into individual nsps including replicase proteins (④). The replicase proteins establish replication and transcription complex (RTC) to initiate the synthesis of a negative-strand (-) to synthesize a new positive-strand RNA genome (⑤). During replication, subgenomic RNAs (sgRNAs) are generated, which encode structural and accessory proteins (⑥). The translated structural proteins migrate to the endoplasmic reticulum (ER) and Golgi apparatus membranes (⑦), where the viral virion is assembled with the encapsidated genomic RNAs (⑧). Finally, the progeny virions are released from the host cell by exocytosis (⑨, ⑩). Of note, the S1-S2 boundary region of S protein in SARS-CoV-2 and MERS-CoV is cleaved by furin during virion maturation before the release of progeny virions, whereas SARS-CoV remains in an unprocessed form.

Two distinct mechanisms of SARS-CoV-2 entry have been suggested: direct fusion with the host cell membrane and clathrin-mediated endocytosis (Fig. 1) [30]. For membrane fusion, coronavirus S proteins are processed by host cellular proteases at two separate cleavage sites: the S1–S2 boundary and the S2’ site of the S2 subunit [31]. At the beginning of the entry process, both the S1–S2 boundary and S2’ sites are cleaved by host proteases in the target cells that viruses infect to facilitate membrane fusion [32]. However, in some types of coronaviruses, the S1–S2 boundary site is cleaved by furin in the Golgi apparatus during virion maturation before progeny virions are released. Previously, it was reported that the MERS-CoV S protein undergoes this pre-processing before virion maturation, finally leading to a two-step sequential cleavage: the first cleavage in replicating cells and the second cleavage in infecting cells [33]. In addition, a recent study reported that the SARS-CoV-2 S protein requires a two-step cleavage to be activated, and the multibasic motif containing multiple arginine residues in the S1–S2 site is essential for this reaction. This unique multibasic motif is found in the S proteins of SARS-CoV-2, MERS-CoV, HCoV-OC43, and HCoV-HKU1 [34]. Receptor binding of the viral S protein causes a conformational change in its S1 subunit, which exposes the S2’ cleavage site of the S2 subunit, and is processed by TMPRSS2, which is a type II transmembrane serine protease. Subsequently, a fusion event between the viral envelope and cell membrane results in the release of viral genomic RNA into the cytoplasm [30, 35]. TMPRSS2 has been reported as a key factor facilitating the entry step in different respiratory viruses, including SARS-CoV-2, SARS-CoV [36, 37], MERS-CoV [38], and influenza A and B viruses [39]. If TMPRSS2 is not available or is insufficiently expressed at the binding site, clathrin-mediated endocytosis internalizes the virus particles into the endosome, in which cathepsin L (CTSL) cleaves the two CTSL-specific cleavage sites of the viral S protein, releasing viral RNA into the cytoplasm [40-43]. Owing to its importance in viral entry, S protein mutations can be a selective pressure for new variants. Since January 2020, the World Health Organization (WHO) has designated five variants of concern (VOC) for SARS-CoV-2, namely Alpha, Beta, Gamma, Delta, and the currently circulating Omicron [44]. Numerous mutations in the S protein show increased transmissibility and virulence between subsequent VOCs. Furthermore, mutations in the RBD of the S1 subunit can increase the binding affinity to cellular receptors and potentially confer resistance to post-vaccination sera [45]. The N-terminal domain (NTD) of the S1 subunit has a supersite to which most NTD-specific neutralizing antibodies can bind; this suggests that mutations in the NTD potentially serve as a selective pressure for SARS-CoV-2 variants [46]. Mutations in some residues of the S2 subunit can cause conformational changes in the S protein complex, altering its stability and thereby affecting viral infectivity [47].

It has been well established that ACE2 is a primary receptor for SARS-CoV-2 infection, and its expression is observed in almost all types of human tissues besides the lung, which is the main infection site. The mRNA levels of human ACE2 vary in different tissues [48]. Recent studies have shown that ciliated and alveolar type 2 (AT2) cells in the lung express both ACE2 and TMPRSS2, and viral infection was detected in the same location [49, 50]. The intestines have been considered as another target organ under infection with SARS-CoV-2 due to gastrointestinal symptoms in some patients [51]. ACE2 mRNA level was highest in the small intestine among the tested organs [48]. Immunohistochemistry results have also shown that ACE2 is highly expressed in enterocytes of the small intestine, along with an abundance of viral antigens [50]. The kidney might be an additional target of SARS-CoV-2 because high expression levels of ACE2 have been detected in the proximal and distal tubules and collecting ducts in the kidney [50]. This is supported by clinical reports that observed acute kidney injuries in some severe COVID-19 cases where the virus was isolated from urine samples [52, 53]. Although the lung is the main infection site and lung inflammation is the key symptom in SARS-CoV-2 pathology, ACE2 expression in the human lung is relatively limited compared to other tissues that highly express ACE2 [48, 54]. A possible explanation was given by Eslami *et al*. [55], who suggested the potential role of several candidate co-receptors in facilitating receptor binding and entry of SARS-CoV-2 based on previous findings: integrin, glucose-regulating protein 78 (GRP78), DPP4, tyrosine-protein kinase receptor UFO (AXL), CD147, neuropilin-1 (NRP1), lectins (CD209L (L-SIGN) / CD209 (DC-SIGN)), vimentin, heparan sulfate, and sialic acid.

APN is expressed in the secretory bronchial epithelium [56]. DPP4 is widely expressed in the human body, including bronchial epithelial cells, alveolar type I and II cells, and activated lymphocytes, in almost all organs [57, 58]. However, the overall expression profiles of coronavirus receptors in tissues and organs are very limited, except for ACE2. The receptors of coronaviruses and their tissue distributions are summarized in Table 1.

### Replication Transcription Complex

After entry into the cells, coronaviruses replicate their genome to form a replication transcription complex (RTC), which comprises nps12 (known as RNA-dependent RNA polymerase, RdRp), nsp7, and nsp8, together with other viral proteins and cofactors (Fig. 1) [59]. Coronavirus RdRp is a key viral protein that catalyzes viral RNA synthesis and requires cofactors for efficient and appropriate RdRp activity. Biochemical analysis revealed that SARS-CoV nsp12 (RdRp) utilizes Mg^2+^ or Mn^2+^ as cofactors to copy its genome, either in the absence or presence of primers [60, 61]. Importantly, SARS-CoV nsp12 (RdRp) forms a triplex structure in the presence of nsp7 and nsp8, thereby initiating RNA synthesis [59, 62]. Nsp7 and nsp8 facilitate the RNA binding of nsp12 and act as primases [59]. Nsp14, which has exoribonuclease (ExoN) and (guanine-N7)-methyltransferase (N7-MTase) activities necessary for proofreading during replication [63], is also associated with the RNA-synthesizing machinery together with nsp10 [59]. Nsp10 of SARS-CoV interacts with nsp14 (ExoN) and nsp16, activating exonuclease activity [64].

The structure of SARS-CoV-2 nsp12 (Rdrp) has recently been identified by several research groups. Similar to SARS-CoV, SARS-CoV-2 nsp12, nsp7, and nsp8 can form complexes with minimal RNA substrates [65, 66]. Mg^2+^ ions required for polymerase activity are present at the catalytic site of the complex [67]. Interestingly, SARS-CoV-2 nsp12 can also utilize Fe–S as a cofactor [68]. A stable nitroxide, TEMPOL (4-hydroxy-2,2,6,6-tetramethylpiperidin-1-oxyl), which disassembles the Fe-S cluster, can effectively inhibit the polymerase activity [68]. Nsp10/14 complex formation has also been reported for SARS-CoV-2 [69]. Nsp10 of SARS-CoV-2 stabilizes the exonuclease of nsp14 for substrate RNA binding, inducing refolding of the nsp14 lid subdomain [70, 71].

Although the nsp10/nsp14 complexes of SARS-CoV, SARS-CoV-2, and MERS-CoV showed similar in vitro FRET-based exonuclease activity [72], the impact of nsp14 on viral replication may differ depending on the coronavirus despite the highly conserved amino acid sequences among coronaviruses. Nsp14 (ExoN) knockout mutants of MHV and SARS-CoV have growth defects that form smaller plaques and hypermutations due to decreased fidelity [73, 74]. In contrast, ExoN activity is critical for MERS-CoV and SARS-CoV-2 replication, and viral progeny cannot be recovered from knockout mutants [75].

**Fig. 2:**
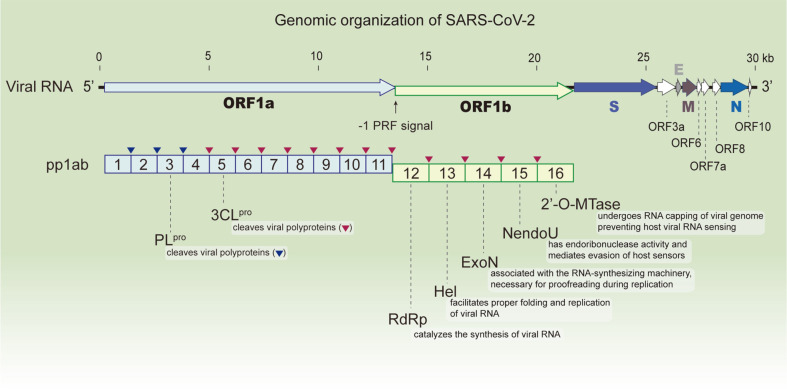
Schematic representation of the genomic organization of SARS-CoV-2 and polyprotein 1ab (pp1ab). SARS-CoV-2 harbors a remarkably large RNA genome (~30 kilobases). It encodes non-structural proteins in the open reading frames 1a and 1b (ORF1a, ORF1b), structural proteins (designated as S, E, M, and N), and other accessory proteins. Two large open reading frames, ORF1a and ORF1b, localized at the 5'-end are translated into polyproteins pp1a and pp1ab. Translation of ORF1b is regulated by a -1 programmed ribosomal frameshift (PRF) signal located upstream of ORF1b generating the pp1ab. The ORF1b contains a replicase gene cluster, which consists of non-structural protein 12 (nsp12) (also known as RNAdependent RNA polymerase, RdRp), nsp13 (helicase, Hel), nsp14 (exoribonuclease/(guanine-N7)-methyltransferase, ExoN/ N7-MTase), nsp15 (nidoviral RNA uridylate-specific endoribonuclease, NendoU), and nsp16 (2′-O-methyltransferase, 2’-OMTase). The pp1ab is cleaved into 16 individual proteins by two viral proteases; papain-like protease (PL^pro^) and 3C-like protease (3CL^pro^, also known as the main protease).

Coronavirus replicase proteins are mainly encoded by the replicase gene cluster (ORF1b), which consists of nsp12 (RdRp), nsp13 (helicase, Hel), nsp14 (ExoN), nsp15 (nidoviral RNA uridylate‐specific endoribonuclease, NendoU), and nsp16 (2′-O-methyltransferase, 2’-O-MTase) (Fig. 2). The translation of these replicase proteins is regulated by a -1 programmed ribosomal frameshift (PRF) signal, which is a secondary RNA pseudoknot structure (approximately 100 bp in length) located between nsp11 and nsp12. When ribosomes encounter a -1 PRF signal during the translation of viral proteins, the ribosome shifts the ribosome reading frame back to one nucleotide and continues the translation of nsp12–16 [76, 77]. Thus, frameshifting efficiency plays an important role in the translation of replicase gene clusters and the subsequent replication of genomic and subgenomic RNAs of coronaviruses [78]. The frameshifting ratio of coronaviruses ranges from 8–30% in the mammalian system [78-81]. A higher frameshift ratio (38–70%) was also observed in MHV [82]. Sequences of the frameshifting signals of SARS-CoV-2 are closer to those of SARS-CoV than to MERS-CoV and HCoVs [80]. The frameshifting efficiencies of SARS-CoV (13.8% for SARS-CoV and 16.3% for SARS-CoV-2) were slightly higher than those of MERS-CoV (8.4%) in the mammalian translation system [80]. The similarity of frameshifting signals between SARS-CoV and SARS-CoV-2 has also been confirmed by recently identified three-dimensional structures [79, 83-85].

Although the molecular mechanism of the frameshifting process has been extensively studied, the impact of frameshifting on viral infectivity, propagation, and transmissibility remains unclear [77]. Frameshift inhibitors targeting the -1 PRF of SARS-CoV-2, either by disrupting the RNA structure or inhibiting the host translation machinery, have been recently developed [80, 85-88]. Structural studies on the interactions between these drugs and RNA pseudoknots are important for the development of drugs against *pan*-coronaviruses.

### Cell Fate Determination

**Fig. 3:**
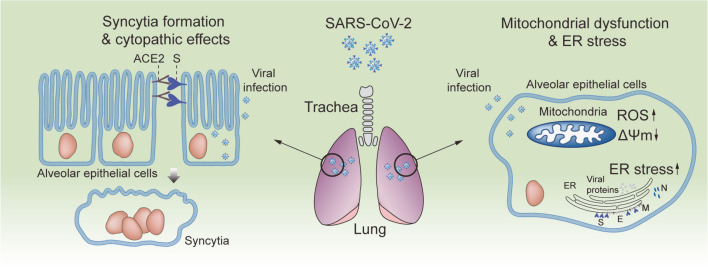
Schematic representation of the lung damage induced by SARS-CoV-2 infection. In the human airway epithelium, SARS-CoV-2 infection induces the formation of syncytia and unique cytopathic effects (CPEs) associated with the clinical symptom of pneumonia. Interaction of the S protein on the cell surface of SARS-CoV-2-infected cells with ACE2 on the cell surface of adjacent cells is a cause of syncytia formation and cell fusion. In the meantime, SARS-CoV-2 infection causes mitochondrial damage, including depolarization of mitochondrial membrane potential (ΔΨm), opening of mitochondrial permeability transition pores, and increasing ROS release, ultimately disrupting mitochondrial homeostasis. Viral proteins, including the S protein and an accessory protein encoded in ORF8, can also induce cellular ER stress modulating innate immunity and inflammatory responses associated with immune evasion.

In the human airway epithelium, SARS-CoV-2 infection lyses cells and forms syncytia, inducing unique cytopathic effects (CPEs) [89, 90]. As illustrated in Fig. 3, syncytia formation occurs when the S protein on the surface of SARS-CoV-2-infected alveolar cells interacts with ACE2 on the surface of adjacent cells [91]. This leads to cell fusion and the occurrence of multinucleated syncytial pneumocytes in the lungs of patients with COVID-19 [89]. Similar to general coronaviruses, a feature of CPE in SARS-CoV-2 is the formation of double-membrane vesicles (DMVs), which contain viral RNA as the intermediate of newly synthesized RNA [92, 93]. In addition, SARS-CoV-2 infection causes aggregation of cellular organelles, vesicles, and virus particles close to the apical surface of ciliated alveolar epithelial cells, generating unique plaque-like CPEs in which multinucleated syncytial cells form a net-like structure [92]. These CPEs might be critical factors that impair the appropriate functions of the alveolar epithelium and are associated with the main clinical symptom of pneumonia. Moreover, significant apoptosis signals were observed in the CPE regions, but necrosis was rarely detected in alveolar epithelial cells [92].

Viral infection can alter a variety of cellular responses that are critical for maintaining homeostasis. ORF3a, which is a conserved accessory protein in coronaviruses, has been reported to have pro-apoptotic activities [94-96]. In addition, the SARS-CoV-2 S protein potentially upregulates the cellular level of reactive oxygen species (ROS) followed by inhibition of the PI3K/AKT/mTOR signaling cascade, leading to autophagy, which is a trigger of cellular inflammation and apoptosis [97]. Another accessory protein of SARS-CoV-2, ORF8, can induce cellular ER stress, which causes inhibition of interferon beta (IFN-β) production and is thus associated with immune evasion [98]. SARS-CoV-2 infection can interrupt mitochondrial homeostasis by causing mitochondrial damage, including depolarization of mitochondrial membrane potential (ΔΨm), opening of mitochondrial permeability transition pores, and increased ROS release [99]. Senescence is an important aging process and a key mechanism in various age-related diseases. Recent studies have revealed that lung cell senescence can be triggered by SARS-CoV-2 S protein and is associated with inflammatory cytokine release [100, 101]. The connection between aging and senescence has been considered an important risk factor for various diseases, including COVID-19. This may explain why the elderly are highly susceptible to infection and show more severe pathological symptoms than the younger population. Diabetes is also a key risk factor for COVID-19, and clinical reports have described that the COVID-19 mortality rate is closely related to diabetes [102-104]. Based on the role of diabetes in COVID-19 pathology, a recent study described a more detailed mechanism of pancreatic beta cell transdifferentiation upon SARS-CoV-2 infection, resulting in reduced expression of insulin and increased production of glucagon and trypsin1 [105]. The cellular responses mentioned above, such as apoptosis, ROS, ER stress, and senescence, have been previously reported for different types of coronaviruses, including SARS-CoV, MERS-CoV, and HCoVs [106]. Thus, it is highly likely that common coronaviruses share mechanisms that modulate host cellular responses, suggesting potential therapeutic targets for broad-spectrum antiviral drugs.

### Virulence Factors associated with SARS-CoV-2 Pathogenicity

Studies to date have shown that when a virus mutates into a new species, the initial toxicity can range from asymptomatic to highly pathogenic; however, specific virulence is difficult to predict. The combination of characteristics of the host, virus, and environment has the potential to determine pathogen virulence [107]. Generally, virulence can be determined by viral structural proteins during host cell entry and by the type of nsps involved in each step of the viral life cycle.

The S protein contributes to viral invasion and virulence through its involvement in the structural integrity of virions and proteolysis [36, 108]. Proteolytic priming of the S protein is mediated either by TMPRSS2 [43, 109] or the cysteine proteases cathepsin B/L [110] for cell fusion- or endocytosis-mediated entry, respectively. The inhibition of these enzymes by small molecules strongly hinders viral entry in vitro [111]. In host cells, cleavage of the SARS-CoV-2 S protein at the furin recognition site of the S1–S2 boundary site is associated with SARS-CoV-2 S-mediated entry, which may affect virulence and tropism [112]. Of the two subdomains of the S protein, the S1 domain contains two relatively independent structures, an N-terminal (S1-NTD) and a C-terminal (S1-CTD) domain [113]. Specifically, within the S1 domain, RBDs in the S1-CTD recognize protein receptors (*e.g.*, ACE2, APN, and DPP4), whereas RBDs in the S1-NTD bind to sugar receptors (*e.g.*, 9-O-Ac-Siac) and evolve faster [114]. In general, protein receptors have a higher affinity for viral attachment than sugar receptors, suggesting that positive selection or relaxed conservation of S1-NTD can be a way to interfere with this binding and reduce the risk of deleterious mutations [115].

E and N proteins, other major structural proteins of SARS-CoV-2, may also contribute to the maintenance of virulence and structural integrity of coronaviruses [116]. Deletion of the E protein or loss of its ion channel activity in SARS-CoV results in attenuation of virulence and alters viral pathogenesis [117, 118]. The C-terminal RNA binding domain (CRBD) and N-terminal RNA binding domain (NRBD) of the N protein are essential for efficient binding to CoV RNA when present inside the host cell; thus, they can be used as a therapeutic drug target [109].

Furthermore, other virulence factors, including nsp1, nsp3, and ORF7a, interfere with the innate immunity of the target host and evade the coronavirus immune system. Nsp1 specifically interacts with the host 40S ribosomal subunit [119], which induces specific degradation of host mRNA and inhibits type I interferon (IFN) production [120].

Nsp3 (also known as PL^pro^) is involved in the cleavage of proteinaceous post-translational modifications of host proteins, which is a mechanism that evades the host's innate immune system. Activation of type I or II IFN signaling induces adenosine diphosphate (ADP)-ribosylation of host proteins. The N-terminal region of full-length nsp3 (PL^pro^), harboring the ADP-ribose (ADPR) domain (known as the macrodomain or Nsp3c), binds to ADP-ribose and can reverse IFN-induced ADP-ribosylation [121, 122]. A recent study revealed that SARS-CoV PL^pro^ (SCoV-PL^pro^) and SARS-CoV-2 PL^pro^ (SCoV2-PL^pro^) differentially regulate host IFN and NF-κB pathways via ubiquitination [123]. Ubiquitination, a post-translational modification process, is of paramount importance in restricting IFN signaling and subsequent expression of various IFN-stimulated genes (ISGs) [124, 125]. IFN activation promotes the expression of interferon-stimulated gene 15 (ISG15), a ubiquitin-like protein, and protein modification by ISG15 (ISGylation), which modifies cellular proteins similar to the ubiquitination process [126]. While SCoV-PL^pro^ mainly targets the ubiquitin chain from substrates, SCoV2-PL^pro^ preferentially cleaves ISG15 from substrates (deISGylation), thereby attenuating type I IFN response [123].

Bone marrow stromal antigen 2 (BST-2) in the host limits the efflux of enveloped viruses and, in turn, internalizes virions through endocytosis [122]. Multiple N-linked glycosylations (N65 and N92) can modify BST-2 at two sites in the extracellular domain [127]. Alterations in these residues are important for antiviral activity. SARS-CoV ORF7a binds directly to BST-2 and inhibits glycosylation, thereby inhibiting BST-2 activity [128]. This evidence suggests that ORF7a, nsp1, and nsp3 play important roles in conferring virulence and thus serve as potential targets for anti-coronavirus drug development.

### Innate Immunity and Immune Evasion

As a first-line defense mechanism, host cells activate innate immune responses to clear invading pathogens and infected cells. To initiate this antiviral mechanism, host cells detect the molecular patterns of components of invading pathogens via pattern recognition receptors (PRRs). Similar to common RNA viruses, cytosolic viral RNA genomes of SARS-CoV-2 are detected by encompassing retinoic acid-inducible gene I (RIG-I) and melanoma differentiation-associated protein 5 (MDA5), the host’s cytosolic RNA sensors, which activate the mitochondrial antiviral signaling protein (MAVS) to proceed with further antiviral responses [129]. If viral entry is mediated by endocytosis, Toll-like receptors (TLR) 3, 7, and 8, as endosomal PRRs, recognize viral genomic RNA [130, 131]. TLR7 has been reported as a critical sensing molecule for several highly pathogenic coronaviruses, such as SARS-CoV, MERS-CoV, and SARS-CoV-2 [130, 132, 133]. In the endosome, activated TLRs recruit Toll-interleukin receptor (TIR) domain-containing adaptor proteins, including myeloid differentiation factor 88 (MyD88), TIR domain-containing adaptor protein (TIRAP), and TIR domain-containing adaptor protein inducing IFN-β-related adapter molecule (TRIF), followed by activation of transcription factors, such as IFN regulatory factor-3 (IRF-3), IRF-7, and nuclear factor kappa light-chain-enhancer of activated B cells (NF-κB), which contribute to the production of cytokines and chemokines [134]. Cytokine storms have been observed in severe cases of patients infected with several coronaviruses, including SARS-CoV, MERS-CoV, and SARS-CoV-2 [135, 136]. In the case of SARS-CoV-2, the key immune cells that contribute to the cytokine storm are monocytes, macrophages, neutrophils, NK cells, dendritic cells, and intraepithelial lymphocytes, which produce pro-inflammatory cytokines and chemokines, including IL-1β, IL-2, IL-6, IL-7, IL-10, TNF-α, IFN-γ, G-CSF, CCL2, and CXCL10 [136].

Meanwhile, coronaviruses have developed immune evasion strategies to facilitate their propagation via different viral components, including various non-structural, structural, and accessory proteins capable of modulating the host IFN signaling pathway [137]. Innate immune evasion strategies can be achieved by targeting the host’s sensor molecules that recognize viral factors and block various downstream antiviral signaling cascades [138]. Representative targets for immune evasion by coronaviruses have been reported extensively: (1) innate sensors such as RIG-I and MDA5, (2) intermediate signaling molecules required for IFN production, (3) IFN signaling molecules, and (4) transcription of ISGs and inflammatory genes [137, 138].

(1) Blocking viral RNA sensing via RIG-I and MDA5: Non-structural proteins (nsp3, nsp4, and nsp6) of common coronaviruses result in the generation of DMVs that shield viral RNA from the host sensors [139-141]. The nsp10-nsp16 complex of coronaviruses modulates viral RNAs by adding a cap at the 5’ end to avoid sensing by RIG-I and MDA5 [142-145]. Under stress conditions, including viral infections, stress granules (SGs), which are cytosolic RNA granules, are generated by wrapping viral RNA and proteins. Accumulated viral RNA in SGs is recognized by RIG-I and MDA5, so that host innate immune responses are stimulated [146]. One strategy to block host viral RNA sensing is to target SG, and different viral factors have been reported depending on the viral subtypes: (i) 4a of MERS-CoV [147], (ii) nsp15 of both SARS-CoV and SARS-CoV-2 [148], and (iii) N of SARS-CoV, SARS-CoV-2, and MERS-CoV [149]. Moreover, SARS-CoV-2 N protein can bind to RIG-I and inhibit the activation of IFN signaling [150].

(2) Targeting signaling pathway for type I IFN production: After viral RNA is recognized, RIG-I/MDA5 initiates subsequent signaling pathways and activates downstream kinases and transcription factors such as IRF3 and NF-κB, which are important for the transcription of type I IFN genes. Secreted type I IFNs can bind to the receptor complex IFNAR1/IFNAR2 on the plasma membrane and then initiate JAK/STAT signaling, in which activated STAT1/2 by phosphorylation recruits IRF9, forming a complex. The STAT1/2/IRF9 complex translocates to the nucleus, facilitating the expression of ISGs [151]. In contrast to SARS-CoV, the SARS-CoV-2 protease nsp3 has a unique substrate preference with ISGylated molecules, thus cleaving ISGylated IRF3 (deISGylation), which is important in IFN production, blocking the host’s innate immunity [123]. In both SARS-CoV and MERS-CoV, nsp3 can inhibit the phosphorylation and subsequent nuclear translocation of IRF3, thereby suppressing IFN production [152, 153]. Moreover, SARS-CoV nsp3 can block the degradation of IκB, an inhibitor of NF-κB, thus inhibiting NF-κB-mediated IFN production [154]. SARS-CoV-2 nsp13 can directly interact with and block the activation of TANK binding kinase 1 (TBK1) and subsequent phosphorylation of IRF3, which is required for IFN gene expression [155]. Similarly, MERS-CoV OFR4a can interact with the TBK1 complex, thereby suppressing IRF3 phosphorylation [156]. ORF9b of SARS-CoV-2 interacts with TOM70, which recruits downstream signaling molecules such as TBK1 and IRF3 to the mitochondria, disrupting further signaling pathways for IFN production [157].

(3) Targeting type I IFN signaling cascades: Nsp14 of SARS-CoV-2 can induce lysosomal degradation of IFN-α receptor 1 (IFNA1), thus disrupting downstream STAT1 phosphorylation [158]. In the case of SARS-CoV, IFNAR1 antagonism is mediated by ORF3a [159].

(4) Inhibiting the transcription of ISGs and inflammatory genes: In both SARS-CoV and SARS-CoV-2, ORF6 binds to Nup98, finally inhibiting the nuclear import of STAT1 and STAT2, which are important factors for the transcription of ISG genes [160, 161]. In addition, several non-structural and accessory proteins of SARS-CoV-2, such as nsp1, nsp6, nsp13, ORF3a, ORF7a, and ORF7b, can inhibit the phosphorylation of STAT1, STAT2, and subsequent interferon stimulation response element (ISRE) promoter activity [155].

### Viral Persistency and Post-Acute Sequelae of SARS-CoV-2 Infection (PASC)

Most patients with COVID-19 recovered after two weeks of SARS-CoV-2 infection. However, some patients with COVID-19 suffer from persistent illness due to systemic hyperinflammatory responses after SARS-CoV-2 infection. These post-acute sequelae of SARS-CoV-2 infection (PASC), so-called 'long COVID', are now under investigation by several groups [162, 163]. Viral persistence is considered a contributor to long COVID and prolonged viral detection of SARS-CoV-2 for several months in the upper respiratory tract, lower respiratory tract, feces, and blood has been reported [164]. Prolonged RNA shedding from several weeks to several months after recovery from acute disease has also been reported for SARS-CoV and MERS-CoV [165-170]. In addition to viral RNA shedding, the SARS-CoV-2 nucleocapsid protein was also found in the intestinal epithelium of a pediatric patient with COVID-19 three months after acute infection [171]. Some patients with COVID-19 were readmitted after pneumonia resolved due to viral persistence [172]. Despite the urgent need to understand viral persistence, few studies have been conducted. Although some studies have reported the establishment of persistent infection with SARS-CoV or SARS-CoV2 in cell lines [173-176], the molecular mechanisms of SARS-CoV-2 persistence have been poorly investigated.

Co-infection with other viruses may lead to viral persistence. In sub-Saharan Africa, where HIV/AIDS is prevalent, co-infection with HIV/SARS-CoV-2 has raised concerns regarding the severity and mortality of COVID-19. Indeed, several large analyses have shown that people infected with HIV have a higher risk of developing severe COVID-19 and dying [177-179]. Recent co-infections with viruses other than HIV, such as influenza viruses (Flu), respiratory syncytial virus (RSV), or adenoviruses, have significantly increased in-hospital mortality [180]. Several case reports from this particular group suggest that immunocompromised patients are more likely to persist, reactivate, or re-infect with the virus, which warrants further investigation. Interestingly, infectious viral particles have been isolated from immunocompromised patients over a period of several weeks to months, with some of them showing recurrence of symptoms [181-185].

## Summary

Since the current COVID-19 pandemic began, tremendous effort has been devoted to understand the virus and the mechanisms of its pathogenesis for the purpose of developing effective therapeutics. The mechanisms of SARS-CoV-2 entry into host cells have been extensively studied as the viral entry step is a promising target to avoid viral infection at the earliest phase. As discussed in the previous section, a key feature of the SARS-CoV-2 entry mechanism is that its S1–S2 boundary region contains a furin cleavage site, which is important for S protein maturation. It is still unclear how furin cleavage-mediated maturation of S proteins affects viral pathogenesis or receptor-binding efficiency. Further investigation into the role of furin cleavage of S proteins is essential to understand the detailed mechanisms of the coronavirus life cycle and help develop effective therapeutics against SARS-CoV-2 and potential new variants. ACE2, which is the key receptor of SARS-CoV-2, is distributed in most tissues, although its expression level varies depending on the tissue type, suggesting a potential reason for multiorgan failure in patients with severe COVID-19. Recently, many reports have shown that a wide range of cellular signaling, metabolism, and immune responses can be modulated by infection with SARS-CoV-2, suggesting potential therapeutic targets for the development of antiviral drugs. To advance the development of more effective antiviral therapeutics, it is important to precisely understand the mechanism of interaction between viral components and host factors, and how the virus-host interaction regulates different cellular responses to finally generate a cellular environment favorable for virus replication. Owing to the error-prone nature of genome replication, SARS-CoV-2 has evolved over time, generating many variants. Numerous studies have reported on the features of SARS-CoV-2 variants [186-196]. How SARS-CoV-2 variants affect viral infectivity and cause different pathogeneses is still poorly understood. To overcome the ongoing emergence of new variants, it is important to understand the mechanisms of viral mutations and identify viral factors that can alter viral infectivity and pathogenesis.

During the last two years, the structure and function of SARS-CoV-2 have been extensively studied. However, most studies have focused on SARS-CoV-2 while other coronaviruses rarely received attention. It is difficult to compare their functions at the molecular level, which differentiates the pathogenesis and distinct features of SARS-CoV-2 from those of other coronaviruses. Thus, further comparative studies of different types of coronaviruses are necessary to gain deeper insights into the development of effective antiviral therapeutics against potential emerging variants.

## Figures and Tables

**Table 1:** Major cellular receptors for coronaviruses.

Cellular receptors	Tissue expression	Virus	Host	Reference for receptor
ACE2^a^	Abundant in the epithelia of enterocytes of the small intestine	SARS-CoV	Human	[89]
	Widely expressed in a variety of tissues, including the lungs	SARS-CoV-2	Human	[43]
		HCoV-NL63	Human	[197]
DPP4^b^	Widely expressed in the human body, including bronchial epithelial cells, alveolar type I and II cells, and activated lymphocytes	MERS-CoV	Human	[28]
9-O-Ac-Sia^c^	Widely displayed on the surfaces of diverse cells and tissues, including the lungs, trachea, intestines, and colon	HCoV-OC43	Human	[5]
		HCoV-HKU1	Human	[4]
APN^d^	Abundant in the epithelia of the kidney, small intestine, and liver Widely expressed in various tissues, including secretory bronchial epithelia	HCoV-229E	Human	[198]
		FCoV	Feline	[199]
		CCoV	Canine	[199]
		TGEV	Porcine	[26]
		PRCV	Porcine	[25]
CEACAM^e^	Widely expressed in diverse human tissues, including the liver, small intestine, and colon, but not in lungs, bronchi, ovaries, or testes	MHV	Murine	[200]

^a^Angiotensin-converting enzyme 2

^b^Dipeptidyl peptidase 4, also known as CD26

^c^9-O-acetylated sialic acid

^d^Aminopeptidase N

^e^Carcinoembryonic antigen-related cell adhesion molecules
